# Virtual reality for delirium prevention in mechanically ventilated ICU patients: a narrative review

**DOI:** 10.3389/fmed.2025.1686453

**Published:** 2025-12-04

**Authors:** Jiaxin Li, Mengyao Su, Yuhao Zhao, Yuxin Zhu, Yuna Hu, Huijie Zhao, Liming Li

**Affiliations:** 1 College of Nursing and Health, Henan University, Kaifeng, China; 2 Department of Critical Care Medicine, Henan Provincial People's Hospital, Zhengzhou, China; 3 Nursing Department, Henan Provincial People's Hospital, Zhengzhou, China

**Keywords:** virtual reality, intensive care unit, mechanical ventilation, delirium, prevention, summarize

## Abstract

**Objective:**

To explore the advantages, applications and shortcomings of virtual reality technology in the prevention of delirium in ICU patients with mechanical ventilation, and to provide references for clinical practice and research in this field.

**Methods:**

This study adopted the narrative review method and systematically retrieved nine databases and related websites including PubMed, Web of Science, Scopus, Embase, Cochrane Library, China Biomedical Literature Service System, China National Knowledge Infrastructure, Wanfang, and VIP database. It aims to collect relevant Chinese and English literature published before April 30, 2025. This study reviews the application, effect and evaluation of virtual reality technology in the prevention of delirium in ICU patients with mechanical ventilation.

**Results:**

Virtual reality technology can effectively alleviate patients’ negative emotions, enhance the interest and compliance of early activities, and have a positive impact on cognitive functions such as working memory by providing immersive experiences, thereby reducing the risk of delirium from multiple dimensions. Existing evidence indicates that this technology has basic safety and feasibility in such critically ill patients. However, current research generally has limitations such as small sample size, lack of support from high-quality randomized controlled trials, relatively single virtual reality content, insufficient personalization, and insufficient validation of the reliability and validity of related usability evaluation tools in the ICU population.

**Conclusion:**

Virtual reality technology has shown significant potential in preventing delirium in ICU patients with mechanical ventilation. Future research should focus on constructing systematic and individualized virtual reality intervention programs, developing and verifying assessment tools suitable for local ICU patients, and further clarifying its long-term efficacy, optimal implementation model and cost-effectiveness through multi-center large-sample studies, so as to promote the standardized application and clinical transformation of this technology.

## 1 Introduction

Delirium is an acute onset of brain dysfunction, mainly manifested as consciousness disorder, distraction, impaired cognitive function and abnormal perception. The state is usually short-lived and fluctuates within a short period of time (1). Mechanical ventilation (MV) refers to a life support technique that provides respiratory support to patients by connecting a ventilator through an artificial airway (tracheal intubation or tracheotomy). Approximately 50 to 70% of ICU patients need to receive MV treatment (2, 3). The incidence of delirium in ICU patients is 29%, but for those receiving MV, it can be as high as 80%, and the mortality rate within 6 months is 34% (4, 5). Compared with ordinary ICU patients, the limited expression and communication barriers caused by intubation often lead to the delirium symptoms of such patients being easily overlooked and missed. Delirium in ICU patients with mechanical ventilation will prolong the time of mechanical ventilation and extubation, increase the mortality rate, and even lead to long-term cognitive dysfunction (6, 7). Among the management strategies for delirium, prevention is the core issue of delirium intervention. Early intervention targeting the risk factors for delirium occurrence can effectively reduce the occurrence of delirium (8). Virtual reality (VR) technology takes computers as the carrier and combines technologies such as artificial intelligence, sensors and computer simulation to provide a multi-information, three-dimensional and dynamic interactive experience, enabling patients to interact in a virtual environment and obtain corresponding feedback from the interaction (9). The ICU patient group is large and diverse. Different patients receive different medical care measures, face different situation demands and physiological and psychological pressures. Therefore, the preventive measures for delirium will also vary. Formulating corresponding VR technology delirium prevention content based on the specific conditions of patients can improve clinical effects and maximize the advantages of VR technology. Therefore, this study reviews the application of VR technology at home and abroad in the prevention of delirium in ICU patients with mechanical ventilation, and explores the different VR contents designed by scholars, with the aim of providing references for clinical workers and researchers to formulate VR technology delirium prevention contents that meet the specific background and needs of ICU patients with mechanical ventilation. In addition, previous studies have rarely mentioned usability evaluation tools for reflecting patients’ satisfaction and acceptance of VR content. Therefore, this article also reviews the usability evaluation tools of VR technology to enable medical staff to better design and improve VR content.

## 2 Methods

This study adopted the narrative review method to conduct a comprehensive and flexible analysis of the application of VR technology in the prevention of delirium in ICU patients with mechanical ventilation (10). This method is particularly suitable for exploring under-researched topics and complex research topics involving multi-dimensional interactions such as technology, clinical care, and patient psychology in this study. It can achieve an overall description and critical analysis of such topics. Unlike systematic reviews that typically focus on a narrow scope, narrative reviews can flexibly integrate research evidence from diverse sources and with different designs, thereby better responding to the comprehensive exploration needs of this study. In addition, this method enables researchers to conduct subjective reviews and critical analyses on the basis of a systematic review of the literature. The comprehensive summaries produced not only have strong readability but are also more likely to be transformed into reference content that has guiding significance for clinical practice.

### 2.1 Establishment of research questions

1. What are the main application forms and contents of VR technology in the prevention of delirium in ICU patients with mechanical ventilation?
2. How effective is the application of VR technology in the prevention of delirium in ICU patients with mechanical ventilation?
3. What are the advantages, disadvantages and challenges of VR technology in this field at present?
4. How can a systematic and effective prevention plan for VR delirium be constructed in the future?

### 2.2 Literature retrieval strategy

To conduct a comprehensive search for relevant literature, this study systematically searched the following nine databases and related websites: PubMed, Web of Science, Scopus, Embase, Cochrane Library, China Biomedical Literature Service System, China National Knowledge Infrastructure (CNKI), Wanfang, and VIP database. The reason for choosing the above databases and literature retrieval platforms is that they have a wide coverage, are both international and local, and can comprehensively reflect the research progress of VR technology in the prevention of ICU delirium in both Chinese and English contexts. In addition to the above, the references were also traced.

The search terms are based on the previous literature review and group discussion, and are conducted in the form of subject terms + free terms. The retrieval period is from the establishment of the databases and related websites to April 30, 2025. For detailed search terms and search strategies taking PubMed as an example, refer to the Supplementary material.

### 2.3 Criteria for literature inclusion and exclusion

#### 2.3.1 Inclusion criteria

(1) Research subjects: Adult patients with mechanical ventilation in the ICU; (2) Intervention measures: Involving the application of VR technology; (3) Research topic: Focus on delirium prevention or related outcomes (such as anxiety, cognitive function, pulmonary rehabilitation, early activity, etc.); (4) Types of literature: including original research, reviews, case reports and guide-type literature; (5) Publication date: From the establishment of the database or literature retrieval platform to April 30, 2025. (6) Language: Chinese and English literature.

#### 2.3.2 Exclusion criteria

(1) Incomplete information or inability to obtain the full text; (2) non-academic literature such as conferences, letters, commentaries, and corrections; (3) translated version or re-publication.

### 2.4 Literature screening

**Figure 1:**
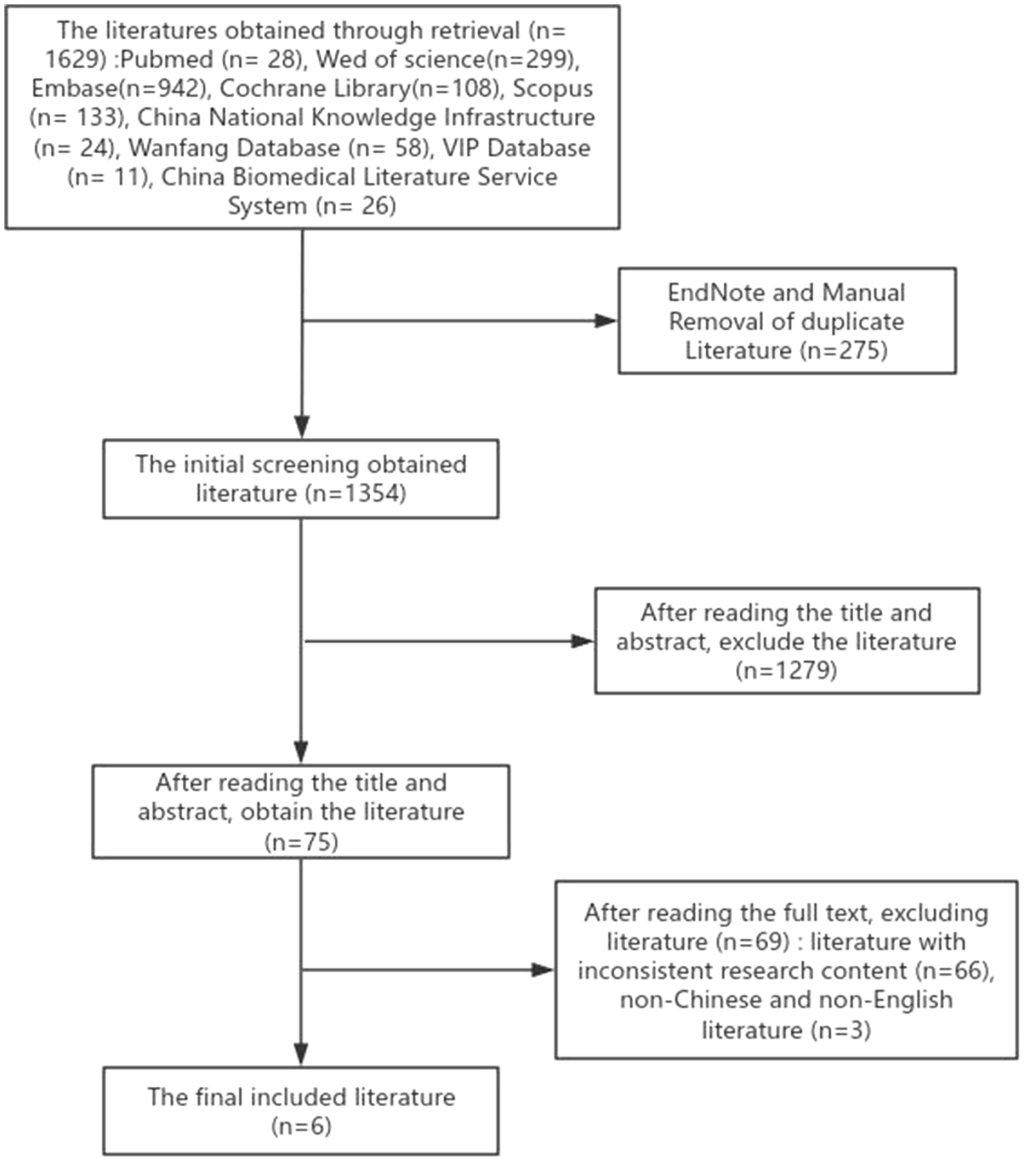
Flowchart of literature screening.

**Figure 2:**
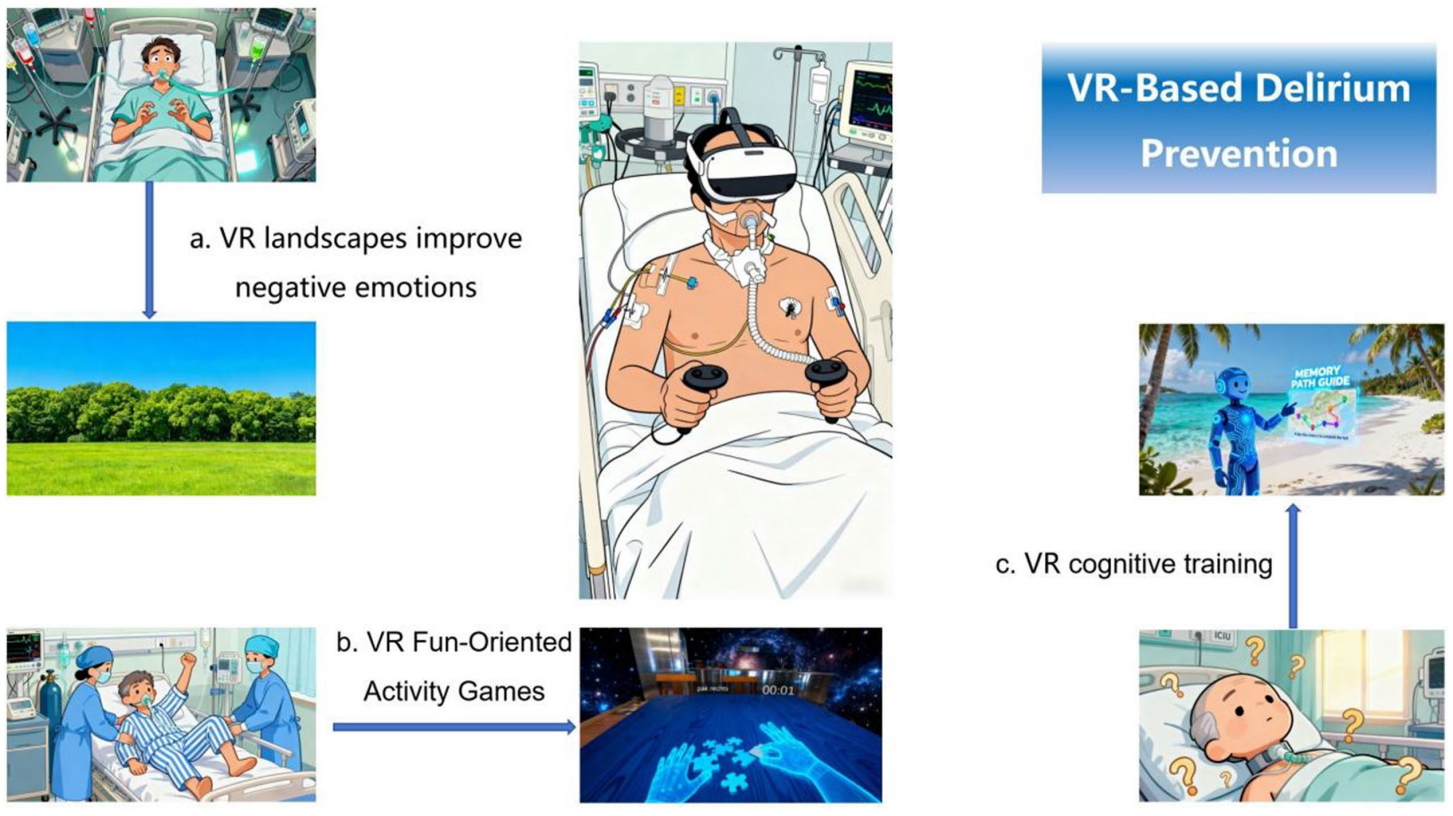
The role of VR technology in the prevention of delirium in patients with mechanical ventilation. **(a)** VR landscapes improve negative emotions: Scholars such as Haley and Wacker (19) pointed out that patients can reduce anxiety by watching videos of outdoor green or blue scenes in VR. **(b)** VR Fun-Oriented Activity Games: Scholars such as de Vries et al. (21) believe that VR jigsaw puzzles can improve patients’ mobility. **(c)** VR cognitive training: Navarra-Ventura et al. (23) found that patients who complete tasks such as route memory guided by virtual avatars in VR scenes of beaches views can improve their cognitive functions.

Two researchers independently carried out literature screening work. If there are any differences of opinion or disputes during the screening process, a third researcher will be invited to participate in the discussion to determine whether the literature will be included. The screening steps are as follows: First, import the obtained literature into the EndNote software and delete the duplicate literature among them to obtain the literature collection after the initial screening. Secondly, by carefully reading the titles and abstracts of the literature, eliminate those that can be clearly judged to be inconsistent with the research topic just from the titles and abstracts, and then obtain the literature after the secondary screening. Next, read the remaining literature in full text to further eliminate those that do not match the research topic of this study in terms of research content and non-Chinese and non-English literature. The literature finally determined to be included in the study. The flowchart of literature screening is shown in Figure 1. Figure 2 summarizes the role of VR technology in the prevention of delirium in patients with mechanical ventilation. The basic information of the literature is shown in Table 1.

**Table 1:** Basic information of the literature.

Author	Publication year/country	Design	Sample	Setting	Main finding(s) relating to disclosure
Blair et al. (18)	2019/The United States	Case Report	One V-V ECMO patient receiving mechanical ventilation	ICU	VR technology can be used to relieve patients’ anxiety without any adverse events, and it is feasible and safe for critically ill patients.
Haley and Wacker (19)	2022/The United States	Quantitative research	Ten patients with mechanical ventilation	Dedicated medical ICU at an academic center and one general ICU at a community hospital	VR intervention with 360° natural scenes can be safely used for patients with mechanical ventilation, with a decrease in anxiety scores and no safety incidents such as tube detachment, supporting further large-scale research.
Chillura et al. (20)	2020/Italy	Case Report	One patient on mechanical ventilation	Transferred from the ICU to the neuro-rehabilitation department for treatment of ICU-acquired weakness	Task training that combines robots and VR is a safe and feasible reinforcement rehabilitation method, which can improve patients’ muscle strength, mobility and functional independence.
de Vries et al. (21)	2025/Netherlands	Quantitative research	Ten patients with mechanical ventilation	ICU	VR games can enhance patients’ enthusiasm and compliance in upper limb activities. The training process is safe and can effectively improve patients’ balance and mobility.
Turon et al. (22)	2017/Spain	Quantitative research	Twenty patients on mechanical ventilation	Mixed medical/surgical ICU	Early neurocognitive stimulation based on VR is feasible, safe and tolerable for critically ill patients. It can effectively stimulate cognitive function and is well-received by patients.
Navarra-Ventura et al. (23)	2021/Spain	Quantitative research	Seventy-two patients with mechanical ventilation	Mixed medical/surgical ICU	Early neurocognitive intervention based on VR can improve short-term and long-term working memory in critically ill patients and may alleviate anxiety and depression symptoms, thus having potential for promotion.

## 3 Current situation of delirium prevention in ICU patients with mechanical ventilation

At present, the prevention of delirium in ICU patients with mechanical ventilation mainly includes etiological prevention, drug prevention, prevention with one or more non-drug measures, and comprehensive management strategies. The treatment of the primary pathogenic mechanism lies in eliminating or alleviating underlying causes such as infection, metabolic disorders, drug poisoning, cerebrovascular diseases, etc., reducing the stimulation of brain function disorders, maintaining the normal function of the nervous system, and thereby preventing the occurrence of delirium. Therefore, in terms of etiological prevention, it is crucial to emphasize the rapid and effective handling of primary diseases and triggers by multidisciplinary teams to curb their development (1). In drug prevention, dexmedetomidine is the only drug proposed and recommended by guidelines for shortening the duration of mechanical ventilation, facilitating early extubation for patients, and reducing the risk of delirium. However, such drugs have side effects such as bradycardia and hypotension (8, 11). Non-pharmaceutical intervention measures such as early activity and sleep management have multiple advantages including high safety, good cost-effectiveness and easy implementation. However, their relatively monotonous and dull nature leads to low enthusiasm and compliance among patients (12). Among the comprehensive management strategies, the representative eCASH strategy (13), ABCDEF strategy (14), and ESCAPE strategy (15) provide a more comprehensive perspective for delirium prevention work. However, they require a large amount of human and time resources, especially in a medical environment with tight human resources, which may be difficult to implement (12, 13).

## 4 VR technology

### 4.1 Overview of VR technology

The integration of VR technology and medicine can be traced back to 1991. M Krueger believes that in the future, integrating VR technology into teaching and training will be a trend in nursing education (16). In the same year, The Lancet published an article discussing the ethical issues of VR, emphasizing the need to pay attention to the application of VR in clinical practice to avoid delaying patients’ opportunities to gain new insights into their diseases (17). VR technology can be classified into four main types based on the way of participation: desktop, immersive, augmented reality and distributed. The characteristics can be summarized into three “is”: Immersion refers to a realistic and immersive feeling, Interaction refers to the interaction between perceiving and operating the environment, and Imagination refers to creativity (9). VR devices are mostly composed of VR headsets and controllers, and some devices are also equipped with headphones. At present, the VRS mainly used in ICU mechanical ventilation patients include Samsung Gear VR Headset1, Samsung Galaxy S7 smartphone coupled with Topmaxions headsets 2, and Evo workstation 33, Oculus Quest 2®22, ENRIC platform (18–23).

### 4.2 Advantages of VR technology in preventing delirium

In previous studies, VR technology has improved patients’ attention by providing immersive and surround experiences and engaging multiple senses (vision, hearing, and kinesthetic). By providing positive environmental stimuli to enhance patients’ emotional states, reduce environmental stress, help patients better control their behaviors and emotions, and establish positive emotional and thinking patterns (24). Secondly, compared with traditional delirium prevention measures, VR technology, by simulating relaxing and pleasant scenarios combined with light music and guiding language, is more vivid and interesting, which helps to stimulate patients’ confidence in recovery, enhance their enthusiasm, and increase their active participation (25). Compared with comprehensive and clustered intervention strategies, VR technology can achieve the effects of multiple non-pharmaceutical intervention measures with a single device, saving time and human resources and improving clinical work efficiency (24).

### 4.3 VR technology evaluation tools

At present, there are mainly four relatively mature tools for evaluating the usability of VR technology. All of them use the Likert Scale to reflect patients’ experiences, attitudes and views on the usability of VR technology. Medical staff can improve and optimize VR content based on the scale scores to provide patients with a better VR experience. For patients with oral intubation or tracheotomy, due to their limited expression ability, when using assessment tools, in addition to marking the score by themselves with a pen, nurses can also assist in marking the score. Patients can confirm by nodding or express the corresponding score to the nurse by gestating with their fingers.

#### 4.3.1 Immersion scale

The Igroup Presence Questionnaire (IPQ) was developed by Schubert et al. (26) and is available in German, English, Dutch and Japanese versions. The English version of the IPQ scale is widely used in the measurement of immersion in VR. This scale consists of 14 items. The first item reflects immersion as a whole and does not belong to any one dimension. The remaining parts can be divided into three dimensions: spatial immersion, sense of participation, and sense of reality. It adopts the Likert 6-level scoring method, with scores ranging from −3 to +3, respectively. The total score ranges from −84 to +84 points. The higher the score, the stronger the immersion. In 2019, scholars Wang et al. (27) translated it into Chinese and conducted reliability and validity tests among 227 adult teachers and students. The Cronhbach’s *α* coefficients ranged from 0.608 to 0.757. Exploratory factors confirmed the three-factor structure of spatial immersion, participation, and realism, which was consistent with the original scale. In 2021, scholars such as Liang et al. (28) revised the Chinese version of the scale among 660 students and tested its reliability and validity. The correlation coefficient was above 0.3, indicating good structural validity and reliability. This scale currently lacks reliability and validity tests in ICU patients and has certain limitations.

#### 4.3.2 Simulated vertigo perception scale

The Simulator Sickness Questionnaire (SSQ) was developed by Kennedy et al. (29) based on the multi-purpose sickness questionnaire used for motion sickness caused by various means of transportation such as cars, ships, and aircraft, and is used to measure vertigo caused by VR scenarios. There are a total of 16 items, divided into three dimensions: nausea, eye movement, and disorientation. Each item is scored on a Likert 4-point scale, ranging from zero to severe, with scores ranging from 0 to 3. The total score ranges from 0 to 235.62 points, and the higher the score, the more severe the vertigo symptoms. Zhang et al. (30) translated it into Chinese in 2022 and conducted reliability and validity tests among 188 college students. The Cronhbach’s *α* coefficient of the scale was 0.75, and the test–retest reliability was 0.76. Because the SSQ questionnaire has a relatively comprehensive range of items and a low probability of missing symptoms, it is widely used. However, on the other hand, the SSQ contains a large number of items, and the results may be exaggerated by irrelevant items, lacking specificity. The reliability and validity tests of this scale in ICU patients are still lacking at present.

#### 4.3.3 Virtual reality motion blur scale

The Virtual Reality Sickness Questionnaire (VRSQ) is derived from the SSQ and is specifically used to assess the sickness symptoms experienced when using VR devices. Kim et al. (31) developed VRSQ in 2018, which involves two dimensions: oculomotor nerve and disorientation. It includes a total of nine items: general discomfort, fatigue, eye fatigue, difficulty focusing, headache, full head, blurred vision, dizziness (with eyes closed), and vertigo. Each item was scored using the Likert 4-level scoring method, ranging from zero to severe, with scores of 0 to 3, respectively. The total score ranged from 0 to 100. The higher the score, the more severe the symptoms. The Cronbach’s *α* coefficient for the eye movement dimension was 0.847, and for the disorientation dimension, it was 0.886, indicating good validity. Josupeit et al. (32) further verified the effectiveness of VRSQ in 2023 by including 244 research subjects. Compared with SSQ, VRSQ contains fewer items and is thus faster and more efficient, but it lacks universality. Future research can further explore the symptoms of vertigo and the universality of assessment tools in different VR environments.

#### 4.3.4 Virtual reality system availability scale

The Development of a virtual reality system usability questionnaire (VRSUQ) was studied and designed by scholars Kim et al. (33) in 2024. This questionnaire consists of three dimensions: validity, efficiency and satisfaction, with a total of 9 items. Each item is divided into 5 levels, with scores ranging from 1 to 5, from strong opposition to very much agreement. The higher the score, the more agreement one agrees with the item. The total score ranges from 0 to 100. The higher the total score, the better the experience and the stronger the usability of the VR system. The overall Cronbach’s *α* value ranged from 0.752 to 0.773, indicating good validity. This scale has fewer items while ensuring complete content. It measures both the experience and the discomfort symptoms, making it more suitable for ICU patients. However, this scale has not yet been localized into Chinese and has not been tested for reliability and validity among patients.

## 5 The application effect of VR technology in the prevention of delirium in ICU patients with mechanical ventilation

### 5.1 Alleviate negative emotions

Negative emotions are a key risk factor for delirium. Relevant studies show that among ICU patients, approximately half are disturbed by negative emotions such as anxiety and depression (34). These emotions not only prolong the patient’s hospital stay but may also increase their risk of death within 2 years after discharge (34). ICU patients with mechanical ventilation are more prone to anxiety and depression due to the physical and psychological stress such as pain and discomfort, communication disorders, physical restraint and thirst caused by intubation, which in turn increases the risk of delirium (35). Therefore, effectively alleviating the negative emotions of such patients is crucial for preventing delirium.

VR technology creates an immersive and relaxing environment through multi-sensory audio-visual stimulation, helping patients divert their attention and reduce anxiety and stress. Blair et al. (18) reported a case of using VR to relieve anxiety in a V-V ECMO intubated patient in an adult ICU. This patient experienced tachycardia and shortness of breath due to anxiety, which affected the blood flow and oxygenation of ECMO. When the effects of conventional drugs and psychological intervention were not satisfactory, the team introduced a customized Samsung Gear VR system. Patients interacted with the virtual Penguin through the controller to experience “Pebbles the Penguin.” The results showed that patients subjectively reported a reduction in anxiety and no adverse events occurred, suggesting that VR can be used as a feasible auxiliary means for anxiety management in critically ill patients in the ICU. To further verify the applicability and efficacy of VR in patients with mechanical ventilation, Haley and Wacker (19) conducted a prospective feasibility study, involving 10 patients with mechanical ventilation and completing a total of 18 VR interventions. Patients watch 360° outdoor green or blue scene videos through head-mounted devices, each time for about 5 min. The study was evaluated using a 100-point Visual Analogue Anxiety Scale. A 100-point Visual Analogue anxiety scale was used for assessment in the study. The results showed that after VR intervention, the median anxiety score of the patients decreased by 8.5 points, and no adverse events such as tube detachment or bed falling occurred during the intervention process. This study confirmed the feasibility and safety of VR in alleviating anxiety in patients with mechanical ventilation, providing a methodological reference for subsequent large-scale research. Analyzing the reasons, immersive experiences offer a powerful distraction, shifting patients’ consciousness from the stressful ICU environment to a pleasant and controllable virtual scene, thereby breaking the vicious cycle of negative thinking. Carefully designed natural scenes or interactive games can trigger positive emotional responses and promote the release of endogenous pleasure substances. In addition, the VR environment endows patients with a certain degree of autonomy and control, which helps to counteract the helplessness brought about by the disease and treatment, thereby improving depressive moods.

Although the sample size of the above-mentioned studies was limited and there was a lack of a control group, they constructed an exploration path for VR technology in the management of negative emotions in ICU, from design and implementation to safety assessment, from both case exploration and feasibility trials. In the future, large-sample randomized controlled trials are still needed to further clarify the effectiveness and mechanism of VR intervention in preventing delirium-related negative emotions.

### 5.2 Facilitate early activities

Patients with long-term mechanical ventilation are prone to circulatory disorders and muscle atrophy due to prolonged bed rest and lack of active activities, which in turn affects brain oxygen supply and increases the risk of delirium (36). Research shows that early activities can promote the recovery of brain and limb functions through moderate physical movement, effectively reducing the incidence of delirium (37). However, problems such as tight human resources and a shortage of rehabilitation therapists in ICU often pose challenges to the systematic implementation of early activities (13–15).

VR technology offers a novel, feasible and attractive solution for early activities in ICU. In a study on patients with severe myasthenia, Chillura et al. (20) reported the rehabilitation of a patient with ICU-acquired frailty. This patient was in a state of tracheotomy. On the basis of the limited effect of conventional physical therapy, they received robot-assisted combined VR training, including lower limb exoskeleton gait training and upper limb virtual task training. After the intervention, the patient progressed from being unable to stand initially to being able to walk with double support. The 6-min walk test distance increased from 6 meters to 47 meters, and the functional independence score also rose from 56 points to 84 points. This case suggests that the combination of VR and robotics can provide critically ill patients with effective, repetitive, and task-oriented training, thereby further promoting functional recovery on the basis of conventional rehabilitation. Vries et al. (21) conducted a feasibility study on upper limb VR rehabilitation for ICU patients, including 10 patients with mechanical ventilation for ≥48 h. They used the Oculus Quest 2® headset for customized jigsaw puzzle game training three times a week, with a target duration of 20 min per session. The results showed that the average actual training time for patients was 13 min per session, and the overall compliance rate reached 60%. No adverse events occurred in the study. The patient satisfaction score was relatively high, and the fatigue level increased but was controllable. At the same time, the Morton mobility index of the participants at the end of the study was higher than that before training, indicating a significant improvement in their balance and mobility. This study verified the feasibility and safety of implementing VR training in ICU patients with mechanical ventilation and provided practical references for formulating personalized rehabilitation plans.

VR technology, with its advantages of immersion, interactivity and gamification, provides an innovative solution to overcome the dullness and lack of motivation of traditional rehabilitation. By creating engaging virtual tasks, it is possible to effectively divert patients’ attention from pain and environmental stress, enhance their intrinsic motivation and training compliance, and thereby facilitate the effective implementation of early activities. The above-mentioned studies still have the limitations of small sample size and the lack of a control group. However, as pioneering work to explore the feasibility of VR limb rehabilitation in the population of patients with mechanical ventilation first, they provide important safety data, implementation plan and methodology for subsequent research, and have high reference significance. In the future, it is recommended to develop an adaptive difficulty system that dynamically adjusts tasks based on the patient’s real-time fatigue and ability level to maintain the best training motivation and intensity.

### 5.3 Improve cognitive impairment

Mechanical ventilation is not only a commonly used life-sustaining method for ICU patients, but also an important risk factor leading to neurocognitive impairment (1). Patients who have received mechanical ventilation for a long time often present with cognitive impairment manifestations such as memory decline and decreased executive function. These impairments are not only independent risk factors for the occurrence of delirium but also one of the predictors of the persistence of delirium. The more severe the degree of cognitive impairment, the higher the risk of delirium in patients (38). Therefore, conducting early cognitive training and neurocognitive stimulation for ICU patients with mechanical ventilation is of great significance for the prevention of delirium.

Turon et al. (22) conducted a feasibility study on 20 patients who received mechanical ventilation in the ICU for no less than 24 h, implementing early neurocognitive intervention through a non-immersive virtual reality system. Patients receive 15 to 20 min of cognitive training during their daily waking period, which includes tasks such as passive observation, selective attention, and working memory. The difficulty gradually increases with the course of treatment. The research results show that this intervention method is feasible and safe for critically ill patients, and the patients can tolerate it well. The patient reported that the experience was easy and interesting, and no adverse events occurred. Although the study did not have a control group, through heart rate variability analysis, it was found that patients showed enhanced activation of brain regions related to attention and executive function in cognitive tasks, suggesting that VR intervention effectively stimulated the brain’s cognitive network and providing a preliminary basis for subsequent research. To further verify the long-term effect of VR cognitive intervention, Navarra-Ventura et al. (23) conducted a prospective randomized controlled trial, dividing 72 ICU patients with mechanical ventilation into an early neurocognitive stimulation group and a conventional treatment group. The intervention group received cognitive training in a virtual reality environment every morning. The scenes included natural landscapes such as wheat fields, beaches, forests and mountain views, accompanied by real environmental sound effects. Patients complete cognitive tasks such as route memory under the guidance of virtual avatars. The study conducted follow-up evaluations on the patients 1 month and 1 year after discharge. The results showed that 1 month after discharge, the working memory performance of the patients who received the intervention was better than that of the control group that did not receive the intervention. Longitudinal analysis shows that there is a significant group main effect on working memory, while the time effect and the interaction between groups and time are not significant, indicating that the improvement effect of early neurocognitive stimulation on working memory shows a continuous positive trend within 1 year after discharge. However, the long-term conclusion of this study is based on a reduced sample size, and its stability awaits further verification by larger-scale research.

VR technology provides immersive cognitive tasks through multiple sensory channels, promoting functional connections and cognitive resource allocation among brain networks. Its interactivity and feedback mechanism help enhance patients’ attention, working memory and executive function, thereby improving cognitive status in the early stage and reducing the risk of delirium. However, the above-mentioned research has not yet clarified key parameters such as the optimal type, initiation timing, duration and intensity of cognitive intervention. Future research should focus on developing a systematic and personalized VR cognitive intervention program to enhance its clinical application effect in preventing delirium in intensive care units.

## 6 The insufficiency of VR technology in the prevention of delirium in ICU patients with mechanical ventilation

### 6.1 Spatial limitations and adverse reactions of VR technology

Research shows that mechanical ventilation is one of the main obstacles affecting patients’ activities (39). In ICU, most patients who require mechanical ventilation, due to their severe conditions, are often in a supine or semi-supine position. Although patients can adjust their positions according to their individual conditions, in the 2024 guidelines for the position management of critically ill patients, it is usually recommended that patients with mechanical ventilation maintain a semi-recumbent or supine position to reduce physical exertion and ensure the effectiveness and safety of respiratory support (40). Therefore, a reasonable VR design should ensure that patients can enjoy a comprehensive immersive experience even when they are bedridden. Especially when VR game devices are used in combination with controllers or rehabilitation equipment, it is necessary to fully consider the space limitations when the patient is bedridden, the physical restraint, and the 360-degree panoramic view inside the headset (41). Consistent with the conclusion pointed out by Rutkowski et al. (42) that although the Kinect system has a low cost, it has high space requirements, and the ideal space for its use is a distance of 1 to 2 meters from the sensor and no direct sunlight, as well as the proposal by Naef et al. (43) that space limitations must be considered when creating VR content, to ensure that patients can watch complete VR surround content without turning their heads or walking. At present, only a few studies have taken into account the special situation of long-term bedridden ICU patients when designing VR technology (44). In addition, the research also found that patients may experience discomfort symptoms such as dizziness and nausea when using VR devices (45), which might be related to the patients’ physiological state or the physical setting of the device screen not matching the patients’ visual focus. However, this reaction is tolerable for most patients and will not cause safety issues (46). Medical staff need to closely monitor the patient’s condition and adjust the usage time according to the patient’s specific state to ensure the comfort and effectiveness of VR intervention.

Future VR content development should focus on barrier-free design. Through collaboration with professional technicians, VR technologies that are more in line with the actual situation of ICU mechanical ventilation patients should be developed. For instance, control schemes based on eye-tracking, voice commands or slight head movements should be developed to replace the reliance on controllers or large-scale limb movements, making them more suitable for mechanical ventilation patients with lower muscle strength. In terms of content and scene design, fast-moving or rotating images that may cause dizziness should be avoided, and sufficient static observation and rest nodes should be provided. In addition, the development of content should be carried out through close collaboration with rehabilitation therapists, critical care experts and clinical engineers to create VR intervention programs that better meet the physical and psychological needs of ICU patients undergoing mechanical ventilation, thereby enhancing the clinical application value and applicability of this technology.

### 6.2 VR content lacks diversity and fails to meet the personalized needs of patients

Current research shows that the application of VR in the prevention of delirium in ICU patients with mechanical ventilation is still relatively limited, mainly concentrated on a few types of landscape videos or interactive games, which is difficult to fully meet the needs of patients. Diverse VR content not only enhances the interest of rehabilitation training but also helps to increase patients’ willingness to participate and their compliance with long-term participation, thereby improving the overall intervention effect (25).

However, contrary to the viewpoint of this study, Locke et al. (47) pointed out that commercially available VR devices already have good usability and a relatively low technical threshold. By reducing latency and minimizing the deviation between the virtual scene and the actual head position of the user, no adverse reactions occurred even when 40% (in line with the national average level of ICU mechanical ventilation beds (48)) of the research subjects were patients with tracheal intubation. This indicates that customizing VR content is not necessarily a necessary condition for the application of VR technology. Medical staff can choose the VR content included in commercially available devices for intervention. Although the existing commercially available VR technologies have certain practicality and their preset contents can improve some outcome indicators of patients to a certain extent, they are still difficult to support long-term and systematic intervention cycles. Especially in the application context of delirium prevention, VR intervention needs to be designed for the multi-factor etiology of delirium, and the content should also be more abundant and flexible. Jawed et al. (41) pointed out in a study that it is not yet clear which VR scene or experience is the most effective. The best personalized visual materials and background music for ICU patients and other elements still need further exploration. Similarly, the research proposal put forward by Naef et al. (49) emphasizes that to maintain patients’ interest and ensure the long-term intervention effect, VR content should be rich and diverse. The team produced a total of 44 videos covering various themes such as natural landscapes, animal worlds, and urban parks. These videos were played through commercially available head-mounted devices during patients’ ICU stays, but not using existing content from the devices. New videos were provided for each intervention to avoid repetitive fatigue.

Therefore, at the technical level, mature commercially available VR devices can be given priority, but their content must be systematically and personalized developed. It is suggested that qualitative research be conducted in the future to gain a deeper understanding of patients’ and medical staff’s experiences and views on VR technology in the prevention of delirium, so as to construct a VR intervention plan that meets clinical needs and reflects patients’ wishes.

## 7 Prospects of VR technology in the prevention of delirium in ICU patients with mechanical ventilation

### 7.1 In the future, it is expected to establish a VR technology delirium prevention program for patients with mechanical ventilation in systematic ICU

VR technology has shown initial potential in preventing delirium in ICU patients with mechanical ventilation. With its immersion, fun and interactivity, it targets the common factors that induce delirium, helping patients relieve anxiety and depression, improve cognitive function and promote early activities, thereby preventing the occurrence of delirium from the source. However, the full-scale promotion of this technology relies on a standardized and highly operational systematic plan as guidance.

Globally, European and American countries have taken the lead in exploring this field, combining VR intervention with the internationally recognized delirium management framework. This includes cluster strategies such as A (assessment, prevention and management of pain), B (spontaneous breathing test and spontaneous arousal test), C (selection and optimization of sedatives), D (assessment and management of delirium), E (early activity), and F (family participation and empowerment) (14). However, at present, most foreign scholars only integrate VR technology with a certain measure and have not yet achieved the integration of multiple delirium prevention measures into VR technology. But the management of delirium requires clustered measures, which will be more effective (1). From the current situation in China, although VR technology shows broad prospects, its application is still in a fragmented exploration stage, lacking a systematic solution based on evidence-based medicine and in line with local clinical practice. This deficiency directly leads to a lack of confidence among clinical workers, especially nursing staff, when facing emerging technologies. They generally expect to receive standardized training before application to master its core application skills (50). By analyzing the reasons, a complete and systematic plan can provide clinical workers with clear operation procedures and evaluation standards, thereby reducing the uncertainty of technology application (51).

Therefore, the core task in the future lies in drawing on international advanced experience while closely integrating with the clinical environment and cultural background of ICU in various countries, and is committed to developing a well-structured and clearly step-by-step prevention plan for VR delirium. This plan should not only specify in detail the applicable population, intervention timing, content selection and duration of VR technology, but also integrate the training of medical staff, patient safety monitoring and emergency response plans for adverse reactions. Through the guidance of systematic plans, the scientificity and standardization of nursing services can be enhanced, maximizing the advantages of VR technology in reducing the incidence of delirium, and effectively promoting technological innovation and practical progress in the field of ICU nursing.

### 7.2 In the future, it is expected that the tools for evaluating the usability of VR technology will be more complete

In the ICU field, the application of VR technology has demonstrated great potential, but its large-scale application is still constrained by system availability issues. System availability refers to the extent to which interactive technologies are efficiently, effectively and satisfactorily utilized by users in a specific environment (52). It is a key factor in evaluating whether its design meets user needs and operational habits (52). In the complex ICU environment, any situation where the operation process is cumbersome, the interface design is not adapted to the patient’s special physiological state, or it is difficult to integrate with the existing medical process may trigger the patient’s resistance, reduce the intervention effect, and hinder medical staff from flexibly adjusting the plan based on real-time feedback, thus failing to fully leverage the clinical value of VR technology (53).

Against this backdrop, it becomes crucial to conduct precise and reliable evaluations of patients’ VR experiences. From a global perspective, researchers in countries such as Germany, the United States, and South Korea have developed some specialized assessment tools. However, as VR medical applications themselves are still in their infancy, most tools have not yet completed sufficient reliability and validity verification in the target patient population, which to a large extent limits their universality and clinical application value (26, 29, 31, 33). This global predicament, as pointed out by Vlake et al. (54), lies in the fact that although the assessment tools for the application of VR technology in ICU have been localized, their reliability and validity remain unresolved. Focusing on the Chinese context, the scales currently relied upon in domestic clinical practice and research mostly originated from abroad. Even though they have been localized and designed to facilitate the filling by intubated patients, their measurement attributes in the special population of mechanical ventilation in Chinese ICU have not yet been strictly verified. Given the rapid advancement of VR technology in China’s medical field, it is particularly urgent to develop localized assessment tools with good reliability and validity. However, due to the limitations of the research period and the lack of practical experience, specialized VR assessment tools that are in line with the background of Chinese medical culture and the characteristics of patients are still in a blank or initial exploration stage. This makes it difficult to precisely quantify the actual effect of VR intervention, thereby hindering the in-depth promotion and optimization of this technology.

With the continuous advancement of technology, the design of assessment tools will be more aligned with the actual clinical needs. Researchers can combine multidisciplinary knowledge to develop a comprehensive scale that can comprehensively measure the application effect of VR technology in the ICU environment. These tools not only cover core dimensions such as immersion and simulated dizziness, but also incorporate considerations of patients’ psychological states, physiological responses and cultural backgrounds to ensure their applicability and accuracy in medical scenarios across different countries.

### 7.3 Looking forward to the further promotion of VR technology in clinical practice in the future

From the perspective of global development trends, the strategic position of VR technology in the medical and health field is becoming increasingly prominent. In 2023, the European Commission released the “Web4.0 and Virtual World Development Initiative,” explicitly stating that it should vigorously promote the standardized application of VR technology in clinical practice and accelerate the process of its transformation from experimental research to clinical practice (55). According to industry analysis and prediction, the global virtual world market size will surge from 27 billion euros in 2022 to over 800 billion euros in 2030 (55). This growth trend fully demonstrates the broad development space and application potential of VR technology in the medical and related industries. Meanwhile, in China, positive progress has also been made in the clinical transformation of VR technology. For instance, scholars such as Xu Jianguang led the formulation of an expert consensus on the application of VR technology in the rehabilitation of sensory-motor and cognitive functions, providing crucial guidance for clinical practice (56, 57).

From an economic perspective, although some medical staff have concerns about the investment cost of VR equipment (58), an overall analysis of the ICU situation shows that patients with mechanical ventilation account for approximately two-thirds of the total ICU patients and are a key group in the prevention and control of delirium (2, 3). Meanwhile, with the continuous decline in the cost of VR technology, its promotion and application in the prevention and control of delirium in ICU not only have significant economic feasibility but also have good clinical benefits (59, 60). In addition, according to practical investigation, the current price range of mainstream VR devices in China mainly focuses on 43.75–562.50 euros, and most of these devices have good system compatibility and can be adapted to multiple medical software platforms. These devices are usually equipped with rich interactive functions, including immersive video experiences and specifically designed cognitive training games, effectively enhancing patients’ engagement and compliance. More importantly, with the popularization of digital content production technology, medical staff can customize exclusive rehabilitation plans based on the individualized needs of patients by using simple video editing tools. For clinical scenarios that require high interactivity, the cost of developing a simple interactive program that meets the needs of delirium prevention is approximately 125.00 euros. Considering the reusable feature of the equipment, its long-term input–output ratio has a distinct advantage. At present, a large amount of audio-visual materials suitable for delirium prevention are circulating on professional platforms. Even conventional 2D video resources can be applied to VR environments through technical conversion, which provides sufficient content support for clinical implementation (60).

Overall, with the gradual reduction of technical costs and the continuous accumulation of clinical evidence, VR technology is expected to become a key link in the delirium prevention and control system for ICU patients with mechanical ventilation, providing strong technical support for improving patient prognosis and enhancing the quality of care.

## 8 Conclusion

VR technology has demonstrated remarkable potential in the prevention of delirium in ICU patients with mechanical ventilation and has received extensive attention and practice both at home and abroad. With its immersive, interactive and interesting features, it can effectively relieve negative emotions, promote early activities of patients, improve cognitive abilities, and reduce the risk of delirium from multiple dimensions. However, this field is still in the exploratory stage on a global scale and generally faces challenges such as inconsistent economic benefits, dizziness reactions, spatial limitations, and insufficient content personalization. In the future, China should base itself on the local ICU background, develop VR content that is suitable for it, draw on international experience, and optimize costs and experiences. Internationally, based on evidence-based research, the integration of multiple measures can be deepened, standardized plans can be constructed, and multi-center studies can be conducted to verify their universality and long-term effectiveness.
