# Supplementary material for: Virtual reality for delirium prevention in mechanically ventilated ICU patients: a narrative review
Source: Front Med (Lausanne). 2025 Dec 4;12:1686453. doi: 10.3389/fmed.2025.1686453 (PMC12679386; doi:10.3389/fmed.2025.1686453)
Supplement: Supplementary file 1 [file Data_Sheet_1.PDF]

Supplementary materials

Search terms

"Virtual reality/Virtual reality Technology/Virtual reality System/Virtual reality Environment/virtual reality Exposure Therapy", "ICU/ Intensive Care Unit/Intensive Care Nursing/Critical Care/Acute and Critical Care/Intensive Care", "Delirium/acute Encephalopathy syndrome/Anxiety/Depression/Pain/Negative Emotions/Pulmonary rehabilitation/Respiratory exercise/respiratory training/cognition/cognitive ability/cognition. "Damage/cognitive impairment/activity/limb movement/early activity/sleep/sleep quality/sleep disorder" are Chinese search terms.

The English search terms are as follows: “Virtual Reality/Reality, Virtual/Virtual Reality Technology/Virtual Reality System/Virtual Reality Environment/Virtual Reality Exposure Therapy/Virtual Reality Immersion Therapy/Virtual Reality Therapy/Reality Therapies, Virtual/Reality Therapy, Virtual/Therapies, Virtual Reality/Therapy, Virtual Reality/Virtual Reality Therapies”

“Intensive Care Units/Intensive Care Unit/Unit, Intensive Care/ICU/Critical Care/Care, Critical/Intensive Care/Care, Intensive/Critical Care Nursing/Nursing, Critical Care/Intensive Care Nursing/Nursing, Intensive Care/Critical Illness/Critical Illnesses/Illness, Critical/Illnesses, Critical/Critically Ill/Critically ill patient*/Critical patient*”

“Delirium/deliri*/Delirium of Mixed Origin/Mixed Origin Delirium/Mixed Origin Deliriums/Anxiety/Angst/Nervousness/Anxiousness/Hypervigilance/Depression/Depressive Symptoms/Depressive Symptom/Symptom, Depressive/Emotional Depression/Depression, Emotional/Pain/Suffering, Physical/Physical Suffering/Physical Sufferings/Sufferings, Physical/Ache/Aches/Negative emotions/Breathing Exercises/Exercise, Breathing/Respiratory Muscle Training/Muscle Training, Respiratory/Training, Respiratory Muscle/Cognition/Cognitions/Cognitive Function/Cognitive Functions/Function, Cognitive/Functions, Cognitive/Cognitive Dysfunction/Cognitive Dysfunctions/Dysfunction, Cognitive/Dysfunctions, Cognitive/Cognitive Disorder/Cognitive Disorders/Disorder, Cognitive/Disorders, Cognitive/Cognitive

Impairments/Cognitive Impairment/Impairment, Cognitive/Impairments, Cognitive/Physical movement/Early activities/Sleep/Sleeping Habits/Sleep Habits/Habit, Sleep/Habits, Sleep/Sleep Habit/Sleeping Habit/Habit, Sleeping/Habits, /Sleeping/Sleep Quality/Qualities, Sleep/Quality, Sleep/Sleep Qualities/Sleep Wake Disorders/Disorder, Sleep Wake/Disorders, Sleep Wake/Sleep Wake Disorder/Wake Disorder, Sleep/Wake Disorders, Sleep/Sleep Disorders/Disorder, Sleep/Disorders, Sleep/Sleep Disorder The retrieval period is from the establishment of the database to April 30, 2025.

The retrieval strategy, taking PubMed as an example

Table 1. Pubmed Search Strategy

Pubmed		Total
#1	((((((((((((Virtual Reality[MeSH Terms]) OR (Reality, Virtual[Title/Abstract])) OR (Virtual Reality Technology[Title/Abstract])) OR (Virtual Reality System[Title/Abstract])) OR (Virtual Reality Environment[Title/Abstract])) OR (Virtual Reality Exposure Therapy[Title/Abstract])) OR (Virtual Reality Immersion Therapy[Title/Abstract])) OR (Virtual Reality Therapy[Title/Abstract])) OR (Reality Therapies, Virtual[Title/Abstract])) OR (Reality Therapy, Virtual[Title/Abstract])) OR (Therapies, Virtual Reality[Title/Abstract])) OR (Therapy, Virtual Reality[Title/Abstract])) OR (Virtual Reality Therapies[Title/Abstract])	11,520
#2	(((((((((((((((((Intensive Care Units[MeSH Terms]) OR (Intensive Care Unit[Title/Abstract])) OR (Unit, Intensive Care[Title/Abstract])) OR (ICU[Title/Abstract])) OR (Critical	371,484

	Care[MeSH Terms])) OR (Care, Critical[Title/Abstract])) OR (Intensive Care[Title/Abstract])) OR (Care, Intensive[Title/Abstract])) OR (Critical Care Nursing[MeSH Terms])) OR (Nursing, Critical Care[Title/Abstract])) OR (Intensive Care Nursing[Title/Abstract])) OR (Nursing, Intensive Care[Title/Abstract])) OR (Critical Illness[MeSH Terms])) OR (Critical Illnesses[Title/Abstract])) OR (Illness, Critical[Title/Abstract])) OR (Illnesses, Critical[Title/Abstract])) OR (Critically Ill[Title/Abstract])) OR (Critically ill patient*[Title/Abstract])) OR (Critical patient*[Title/Abstract])	
#3	((Delirium[MeSH Terms]) OR (deliri*[Title/Abstract])) OR (Delirium of Mixed Origin[Title/Abstract])) OR (Mixed Origin Delirium[Title/Abstract])) OR (Mixed Origin Deliriums[Title/Abstract])) OR (Anxiety[MeSH Terms])) OR (Angst[Title/Abstract])) OR (Nervousness[Title/Abstract])) OR (Anxiousness[Title/Abstract])) OR (Hypervigilance[Title/Abstract])) OR (Depression[MeSH Terms])) OR (Depressive Symptoms[Title/Abstract])) OR (Depressive Symptom[Title/Abstract])) OR (Symptom, Depressive[Title/Abstract])) OR (Emotional Depression[Title/Abstract])) OR (Depression, Emotional[Title/Abstract])) OR (Pain[MeSH Terms])) OR (Suffering, Physical[Title/Abstract])) OR (Physical Suffering[Title/Abstract])) OR (Physical Sufferings[Title/Abstract])) OR (Sufferings, Physical[Title/Abstract])) OR (Ache[Title/Abstract])) OR (Aches[Title/Abstract])) OR (Negative emotions[Title/Abstract])) OR (Breathing Exercises[MeSH Terms])) OR (Exercise, Breathing[Title/Abstract])) OR (Respiratory Muscle	1,552,462

	Training[Title/Abstract])) OR (Muscle Training, Respiratory[Title/Abstract])) OR (Training, Respiratory Muscle[Title/Abstract])) OR (Cognition[MeSH Terms])) OR (Cognitions[Title/Abstract])) OR (Cognitive Function[Title/Abstract])) OR (Cognitive Functions[Title/Abstract])) OR (Function, Cognitive[Title/Abstract])) OR (Functions, Cognitive[Title/Abstract])) OR (Cognitive Dysfunction[MeSH Terms])) OR (Cognitive Dysfunctions[Title/Abstract])) OR (Dysfunction, Cognitive[Title/Abstract])) OR (Dysfunctions, Cognitive[Title/Abstract])) OR (Cognitive Disorder[Title/Abstract])) OR (Cognitive Disorders[Title/Abstract])) OR (Disorder, Cognitive[Title/Abstract])) OR (Disorders, Cognitive[Title/Abstract])) OR (Cognitive Impairments[Title/Abstract])) OR (Cognitive Impairment[Title/Abstract])) OR (Impairment, Cognitive[Title/Abstract])) OR (Impairments, Cognitive[Title/Abstract])) OR (Physical movement[Title/Abstract])) OR (Early activities[Title/Abstract])) OR (Sleep[MeSH Terms])) OR (Sleeping Habits[Title/Abstract])) OR (Sleep Habits[Title/Abstract])) OR (Habit, Sleep[Title/Abstract])) OR (Habits, Sleep[Title/Abstract])) OR (Sleep Habit[Title/Abstract])) OR (Sleeping Habit[Title/Abstract])) OR (Habit, Sleeping[Title/Abstract])) OR (Habits, Sleeping[Title/Abstract])) OR (Sleep Quality[MeSH Terms])) OR (Qualities, Sleep[Title/Abstract])) OR (Quality, Sleep[Title/Abstract])) OR (Sleep Qualities[Title/Abstract])) OR (Sleep Wake Disorders[MeSH Terms])) OR (Disorder, Sleep Wake[Title/Abstract])) OR (Disorders, Sleep Wake[Title/Abstract])) OR (Sleep Wake Disorder[Title/Abstract])) OR (Wake Disorder,	

	Sleep[Title/Abstract])) OR (Wake Disorders, Sleep[Title/Abstract])) OR (Sleep Disorders[Title/Abstract])) OR (Disorder, Sleep[Title/Abstract])) OR (Disorders, Sleep[Title/Abstract])) OR (Sleep Disorder[Title/Abstract])	
#4	("1000/1/1"[Date - Publication] : "2025/04/30"[Date - Publication])	38,875,278
#5	#1 AND #2 AND #3 AND #4	28
