# Supplementary material for: Virtual reality for delirium prevention in mechanically ventilated ICU patients: a narrative review
Source: Front Med (Lausanne). 2025 Dec 4;12:1686453. doi: 10.3389/fmed.2025.1686453 (PMC12679386; doi:10.3389/fmed.2025.1686453)
Supplement: Supplementary file 2 [file Table_1.DOCX]

**Supplementary materials**

**Search terms**

"Virtual reality/Virtual reality Technology/Virtual reality System/Virtual reality Environment/virtual reality Exposure Therapy", "ICU/ Intensive Care Unit/Intensive Care Nursing/Critical Care/Acute and Critical Care/Intensive Care", "Delirium/acute Encephalopathy syndrome/Anxiety/Depression/Pain/Negative Emotions/Pulmonary rehabilitation/Respiratory exercise/respiratory training/cognition/cognitive ability/cognition. "Damage/cognitive impairment/activity/limb movement/early activity/sleep/sleep quality/sleep disorder" are Chinese search terms.

The English search terms are as follows: “Virtual Reality/Reality, Virtual/Virtual Reality Technology/Virtual Reality System/Virtual Reality Environment/Virtual Reality Exposure Therapy/Virtual Reality Immersion Therapy/Virtual Reality Therapy/Reality Therapies, Virtual/Reality Therapy, Virtual/Therapies, Virtual Reality/Therapy, Virtual Reality/Virtual Reality Therapies”

“Intensive Care Units/Intensive Care Unit/Unit, Intensive Care/ICU/Critical Care/Care, Critical/Intensive Care/Care, Intensive/Critical Care Nursing/Nursing, Critical Care/Intensive Care Nursing/Nursing, Intensive Care/Critical Illness/Critical Illnesses/Illness, Critical/Illnesses, Critical/Critically Ill/Critically ill patient*/Critical patient*”

“Delirium/deliri*/Delirium of Mixed Origin/Mixed Origin Delirium/Mixed Origin Deliriums/Anxiety/Angst/Nervousness/Anxiousness/Hypervigilance/Depression/Depressive Symptoms/Depressive Symptom/Symptom, Depressive/Emotional Depression/Depression, Emotional/Pain/Suffering, Physical/Physical Suffering/Physical Sufferings/Sufferings, Physical/Ache/Aches/Negative emotions/Breathing Exercises/Exercise, Breathing/Respiratory Muscle Training/Muscle Training, Respiratory/Training, Respiratory Muscle/Cognition/Cognitions/Cognitive Function/Cognitive Functions/Function, Cognitive/Functions, Cognitive/Cognitive Dysfunction/Cognitive Dysfunctions/Dysfunction, Cognitive/Dysfunctions, Cognitive/Cognitive Disorder/Cognitive Disorders/Disorder, Cognitive/Disorders, Cognitive/Cognitive Impairments/Cognitive Impairment/Impairment, Cognitive/Impairments, Cognitive/Physical movement/Early activities/Sleep/Sleeping Habits/Sleep Habits/Habit, Sleep/Habits, Sleep/Sleep Habit/Sleeping Habit/Habit, Sleeping/Habits, /Sleeping/Sleep Quality/Qualities, Sleep/Quality, Sleep/Sleep Qualities/Sleep Wake Disorders/Disorder, Sleep Wake/Disorders, Sleep Wake/Sleep Wake Disorder/Wake Disorder, Sleep/Wake Disorders, Sleep/Sleep Disorders/Disorder, Sleep/Disorders, Sleep/Sleep Disorder The retrieval period is from the establishment of the database to April 30, 2025.

**The retrieval strategy, taking PubMed as an example**

**Table 1. Pubmed Search Strategy**

| **Pubmed** | | **Total** |
| --- | --- | --- |
| #1 | ((((((((((((Virtual Reality[MeSH Terms]) OR (Reality, Virtual[Title/Abstract])) OR (Virtual Reality Technology[Title/Abstract])) OR (Virtual Reality System[Title/Abstract])) OR (Virtual Reality Environment[Title/Abstract])) OR (Virtual Reality Exposure Therapy[Title/Abstract])) OR (Virtual Reality Immersion Therapy[Title/Abstract])) OR (Virtual Reality Therapy[Title/Abstract])) OR (Reality Therapies, Virtual[Title/Abstract])) OR (Reality Therapy, Virtual[Title/Abstract])) OR (Therapies, Virtual Reality[Title/Abstract])) OR (Therapy, Virtual Reality[Title/Abstract])) OR (Virtual Reality Therapies[Title/Abstract]) | 11,520 |
| #2 | ((((((((((((((((((Intensive Care Units[MeSH Terms]) OR (Intensive Care Unit[Title/Abstract])) OR (Unit, Intensive Care[Title/Abstract])) OR (ICU[Title/Abstract])) OR (Critical Care[MeSH Terms])) OR (Care, Critical[Title/Abstract])) OR (Intensive Care[Title/Abstract])) OR (Care, Intensive[Title/Abstract])) OR (Critical Care Nursing[MeSH Terms])) OR (Nursing, Critical Care[Title/Abstract])) OR (Intensive Care Nursing[Title/Abstract])) OR (Nursing, Intensive Care[Title/Abstract])) OR (Critical Illness[MeSH Terms])) OR (Critical Illnesses[Title/Abstract])) OR (Illness, Critical[Title/Abstract])) OR (Illnesses, Critical[Title/Abstract])) OR (Critically Ill[Title/Abstract])) OR (Critically ill patient*[Title/Abstract])) OR (Critical patient*[Title/Abstract]) | 371,484 |
| #3 | (((((((((((((((((((((((((((((((((((((((((((((((((((((((((((((((((((((((Delirium[MeSH Terms]) OR (deliri*[Title/Abstract])) OR (Delirium of Mixed Origin[Title/Abstract])) OR (Mixed Origin Delirium[Title/Abstract])) OR (Mixed Origin Deliriums[Title/Abstract])) OR (Anxiety[MeSH Terms])) OR (Angst[Title/Abstract])) OR (Nervousness[Title/Abstract])) OR (Anxiousness[Title/Abstract])) OR (Hypervigilance[Title/Abstract])) OR (Depression[MeSH Terms])) OR (Depressive Symptoms[Title/Abstract])) OR (Depressive Symptom[Title/Abstract])) OR (Symptom, Depressive[Title/Abstract])) OR (Emotional Depression[Title/Abstract])) OR (Depression, Emotional[Title/Abstract])) OR (Pain[MeSH Terms])) OR (Suffering, Physical[Title/Abstract])) OR (Physical Suffering[Title/Abstract])) OR (Physical Sufferings[Title/Abstract])) OR (Sufferings, Physical[Title/Abstract])) OR (Ache[Title/Abstract])) OR (Aches[Title/Abstract])) OR (Negative emotions[Title/Abstract])) OR (Breathing Exercises[MeSH Terms])) OR (Exercise, Breathing[Title/Abstract])) OR (Respiratory Muscle Training[Title/Abstract])) OR (Muscle Training, Respiratory[Title/Abstract])) OR (Training, Respiratory Muscle[Title/Abstract])) OR (Cognition[MeSH Terms])) OR (Cognitions[Title/Abstract])) OR (Cognitive Function[Title/Abstract])) OR (Cognitive Functions[Title/Abstract])) OR (Function, Cognitive[Title/Abstract])) OR (Functions, Cognitive[Title/Abstract])) OR (Cognitive Dysfunction[MeSH Terms])) OR (Cognitive Dysfunctions[Title/Abstract])) OR (Dysfunction, Cognitive[Title/Abstract])) OR (Dysfunctions, Cognitive[Title/Abstract])) OR (Cognitive Disorder[Title/Abstract])) OR (Cognitive Disorders[Title/Abstract])) OR (Disorder, Cognitive[Title/Abstract])) OR (Disorders, Cognitive[Title/Abstract])) OR (Cognitive Impairments[Title/Abstract])) OR (Cognitive Impairment[Title/Abstract])) OR (Impairment, Cognitive[Title/Abstract])) OR (Impairments, Cognitive[Title/Abstract])) OR (Physical movement[Title/Abstract])) OR (Early activities[Title/Abstract])) OR (Sleep[MeSH Terms])) OR (Sleeping Habits[Title/Abstract])) OR (Sleep Habits[Title/Abstract])) OR (Habit, Sleep[Title/Abstract])) OR (Habits, Sleep[Title/Abstract])) OR (Sleep Habit[Title/Abstract])) OR (Sleeping Habit[Title/Abstract])) OR (Habit, Sleeping[Title/Abstract])) OR (Habits, Sleeping[Title/Abstract])) OR (Sleep Quality[MeSH Terms])) OR (Qualities, Sleep[Title/Abstract])) OR (Quality, Sleep[Title/Abstract])) OR (Sleep Qualities[Title/Abstract])) OR (Sleep Wake Disorders[MeSH Terms])) OR (Disorder, Sleep Wake[Title/Abstract])) OR (Disorders, Sleep Wake[Title/Abstract])) OR (Sleep Wake Disorder[Title/Abstract])) OR (Wake Disorder, Sleep[Title/Abstract])) OR (Wake Disorders, Sleep[Title/Abstract])) OR (Sleep Disorders[Title/Abstract])) OR (Disorder, Sleep[Title/Abstract])) OR (Disorders, Sleep[Title/Abstract])) OR (Sleep Disorder[Title/Abstract]) | 1,552,462 |
| #4 | ("1000/1/1"[Date - Publication] : "2025/04/30"[Date - Publication]) | 38,875,278 |
| #5 | #1 AND #2 AND #3 AND #4 | 28 |
